# From Breastfeeding to Support in Mothers’ Feeding Choices: A Key Role in the Prevention of Postpartum Depression?

**DOI:** 10.3390/nu16142285

**Published:** 2024-07-16

**Authors:** Angelica Dessì, Gaia Pianese, Paolo Mureddu, Vassilios Fanos, Alice Bosco

**Affiliations:** Department of Surgical Sciences, University of Cagliari and Neonatal Intensive Care Unit, AOU Cagliari, 09124 Cagliari, Italy; pianesegaia@gmail.com (G.P.); paolomureddu327@gmail.com (P.M.); vafanos@tiscali.it (V.F.); alice.bosco@unica.it (A.B.)

**Keywords:** neonatal nutrition, postpartum depression, breastfeeding support

## Abstract

The postpartum period represents a critical phase of profound transition for women. This timeframe encompasses the physical recuperation associated with childbirth, the intricate psychosocial adjustments inherent in assuming the role of motherhood and also important alterations in steroid and peptide hormones. Hence, as women navigate the reconfiguration of relationships and strive to address the diverse needs of their infants and family members, they concurrently grapple with dramatic transformations which are characteristic of the postpartum phase. In fact, relevant prevalence ranges are reported for maternity blues, a mild condition characterized by self-limited and transient depressive symptoms, but also a well-established risk factor for more serious postpartum mood disorders, such as depression (PPD), with an incidence of 10–15%. Unlike in the US, at the European level, there are no concrete recommendations for the routine integration of the assessment of the mother’s emotional state by healthcare professionals, with a considerable risk of underdiagnosing or undertreating these conditions. In this regard, there is a growing body of scientific evidence on the important role of breastfeeding in reducing the risk of PPD and also of the importance of mothers’ compliance with this practice. Indeed, sucking the baby regulates the circadian rhythm of the HPA axis and, together with the action of prolactin, the stress response is decreased. In addition, other positive consequences of breastfeeding, which are inversely correlated with the onset of PPD, include the regulation of sleep and waking patterns for mother and baby, the improvement of the mother’s self-efficacy and her emotional involvement. It should also be considered that the request for support for breastfeeding can often conceal a request for support for motherhood itself and for the mother’s emotional well-being. It therefore emerges that the personnel involved in primary pediatric care to provide adequate support in the transition to motherhood must support mothers in their breastfeeding choices, whether breastfeeding or formula feeding, so that each choice is made conscientiously and serenely. Therefore, neonatal feeding assumes a decisive role, since if, on the one hand, it regulates specific neurohormonal pathways that are protective for maternal emotional well-being (breastfeeding), on the other hand, support in mothers’ breastfeeding choices, even in the case of formula feeding, means validating their being mothers in the absence of judgement and counteracting any feelings of inadequacy, conditions that are inversely correlated to DPP.

## 1. Introduction

The postpartum period is the period which starts an hour after childbirth and the expulsion of the placenta and lasts from six to eight weeks after that. It signifies a crucial phase in a woman’s life. During this time, the body undergoes significant changes, with the uterus regressing and physiological systems reverting to a non-pregnant state. However, the postpartum period is more than a mere physiological recovery; it involves intricate psychosocial adjustments. These include shifts in parental responsibilities [1,2], changes in family dynamics [3,4] and alterations in self-perception and body image [1,4,5], many of which extend beyond the initial six to eight weeks.

The year following childbirth emerges as a period where women are particularly susceptible to health challenges. This vulnerability arises from the ongoing adjustments required for caring for a newborn and managing the psychosocial changes, in addition to the physical recuperation from childbirth. Research underscores the impact of unmanaged postpartum stresses, leading to anxiety [6], fatigue [7] and decreased self-care [8]. Furthermore, these stresses correlate with a higher risk of physical and mental illnesses, including postpartum depression [9,10]. Consequently, maternal mental and physical health issues amplify the risk of adverse outcomes for the entire family, such as early breastfeeding interruptions, negative mother–child assessments, delayed language development in the child, a compromised mother–child bond, reduced childhood vaccinations and increased behavioral problems in the child [11,12,13].

Moreover, there is now scientific evidence on the delicate role of breastfeeding in reducing the risk of PPD [14], but also on the importance of mothers’ compliance with this practice [15]. In addition, the call for support for breastfeeding can often conceal a request for support for motherhood itself and the emotional well-being of the mother [16].

Thus, the delicate role of early nutrition within a health promotion strategy emerges not only for the well-being of the baby, thanks to the multiple and unique properties of breast milk, but also in creating his or her bond with the mother, promoting her mental well-being.

Despite these interconnected factors, there remains a noticeable gap in the care provided to women during this transitionary period, necessitating comprehensive actions to support women’s postpartum health, especially if we take into account that, at the European level, there are no concrete recommendations for the routine integration of the assessment of the mother’s emotional state by healthcare professionals, with a considerable risk of undervaluing maternal mental well-being and its consequences [17].

This review seeks to shed light on the importance of breastfeeding support in preventing PPD by carefully examining the specific needs of women after childbirth.

## 2. What Happens in the Postpartum Period: The Origin of PPD 

The postpartum period can be characterized by a particular euphoria, potentially attributable to the release of dopamine in the brain, mediated by oxytocin [18]. Following delivery, oxytocin levels in the bloodstream surge three to four times, potentially coinciding with elevated oxytocin levels in the brain. This increase in oxytocin may be correlated with heightened dopamine release [19]. Oxytocin, released during skin-to-skin contact post-birth, fosters interaction and attachment between mother and child [20]. The pro-social and pro-attachment effects of oxytocin may explain the maternal inclination towards family reunions. Furthermore, oxytocin, released in response to touch, warmth and kinship between mother and child, carries significant anti-stress effects, potentially contributing to the desire for solitude and quiet meditation [21].

The feelings of euphoria and transformation post-childbirth align with remarkably elevated brain levels of catecholamines, oxytocin and dopamine, making the childbirth brain function uniquely exceptional. Oxytocin not only stimulates dopamine release but also activates the serotonin pathway, creating a reciprocal relationship [22,23]. According to the Karolinska Personality Scale, women exhibit increased levels of social interaction behavior and reduced anxiety after childbirth [24]. The release of endogenous oxytocin orchestrates neurobiological processes crucial for childbirth, facilitating the transition to motherhood and contributing to the profound psychological experience of labor.

However, the role of some of these hormones, such as oxytocin and other catecholamines, also seems crucial in the onset of PPD [25,26,27,28,29,30,31,32]. According to scientific research, in the case of PPD, the main players are certain catecholamines (serotonin) [25,26], endocrine hormones, in particular oxytocin, reproductive hormones [25,28,29], stress hormones, in particular those of the hypothalamic–pituitary–adrenal (HPA) [25,28,29,30] axis and inflammatory responses [25,31,32]. Indeed, according to the unified model of PPD recently proposed by Levin et al. [25], pregnancy is characterized by an excessive production of quinolinic acid (QUIN), a potent agonist of the N-methyl-D-aspartate glutamate receptor (NMDAR). Everything originates from the metabolization of the amino acid tryptophan, which is degraded to a small extent (less than 5%) into serotonin and for the remainder (more than 95%) into kynurenine, together with its degradation products, quinolinic acid and NAD+, which includes several neurotoxins, and kinurenic acid, a neuroprotective factor. Several factors are responsible for this overproduction, the main ones being alterations in the immune system during different stages of pregnancy, stress and certain reproductive hormones such as estradiol. QUIN acts not only as an agonist of the glutamate NMDR receptor but also as a blocker of its transporter, leading to numerous neurotoxic effects responsible for the main depressive symptoms, resulting in anhedonia, loss of energy and increased tiredness, increased aimless physical activity or slowed movement or speech, feelings of worthlessness or guilt and lack of interest in the child. The overproduction of QUIN also leads to a depletion of serotonin and thus melatonin, with repercussions on appetite, learning, memory, pain and sleep [25].

A possible protective factor is represented by oxytocin, thanks to its anti-inflammatory properties and its action of inhibiting the HPA stress axis, the release of which, although always influenced by the kynurenine metabolism, is favored by breastfeeding and support. In fact, the kynurenine pathway produces NAD+, the precursor to the CD38-ADP cyclic ribose pathway (cADPR), a fundamental mechanism for oxytocin secretion and the immune response. 

The hypothesis of the unified model of PPD shows how this pathology is the result of a set of interconnected alterations. In fact, although initially the QUIN and NAD+ pathway contributes to the release of oxytocin as a protective factor, later on, an imbalance of CD38-cADPR may occur in the various tissues, which leads to a decrease in the secretion of oxytocin itself. Furthermore, the heterogeneity of PPD and its onset could be determined, not only by environmental factors, but also by individual differences, also caused by genetic polymorphisms, in the expression of all of these components [25].

Despite the comprehensiveness of the proposed PPD models, studies conducted with functional magnetic resonance imaging in women during late pregnancy and up to 18 months postpartum have shown with certainty the presence of significant, often lateralized, changes in neural activity in PPD. These changes affect brain regions that are crucial for self-regulation, empathy and emotion both in situations of complete maternal rest and in the maternal response to care [33].

## 3. The Gravity of the Issue: Prevalence of PPD

PPD is included in the category of perinatal mental illnesses, which are defined as psychiatric disorders that predominantly manifest during pregnancy and up to one year postpartum. These disorders may range in severity, with some presenting as mild depression and others as psychosis. Nevertheless, it is not uncommon for women to experience a variety of physical and emotional difficulties in the first week following childbirth, which are collectively referred to as “postpartum blues” or “baby blues”. These conditions are characterized by a variety of symptoms, including mood swings, irritability, anxiety, insomnia, crying, appetite loss and restlessness, with the prevalence rates highly variable, ranging from 26% to 84%. This is due to the absence of universal diagnostic criteria [34]. Postpartum blues is a common and transient condition that is generally untreated, but it is important to recognize it because it is a risk factor for PPD [35]. 

PPD, on the other hand, is a major depressive disorder and is therefore characterized by a depressed mood or loss of interest or pleasure in activities for at least two weeks. Sleep and appetite disturbances, loss of energy, feelings of worthlessness or guilt, decreased concentration and thoughts of suicide may also be present. What makes the diagnosis of PPD complex is the normal occurrence in the postnatal period of changes in sleep and appetite patterns and the presence of excessive fatigue. One of the widely used validated diagnostic methodologies is the Edinburgh Postnatal Depression Scale, a 10-item questionnaire, the score of which, if ≥12, is indicative of probable PPD that needs to be confirmed with a more rigorous assessment in the context of a clinical interview [34,35]. Regarding the prevalence of this disorder, the results of a systematic literature review in 2018 reported prevalence levels of PPD of 17% among healthy mothers without a previous history of depression, regardless of the type of diagnostic tool used. On the other hand, statistical differences in prevalence emerged between different geographical regions, with the Middle East recording the highest prevalence (26%), while a lower prevalence was observed in Europe (8%) [36]. This review analyzed a specific group of healthy mothers who gave birth to healthy children with no history of mental illness, including PPD, showing similar levels of PPD prevalence compared to mothers with a history of psychiatric illness [36]. More recent data from a systematic review in 2022 showed a high prevalence of PPD, especially in developing countries. In fact, a pooled prevalence of 14% emerged in all studies, but with important variations from country to country (from 5.0% to 26.32%). As many as nine risk factors were also highlighted, including previous depressive problems, both in pregnancy and not, as well as gestational diabetes mellitus, women giving birth to males and epidural anesthesia during childbirth [37]. Recent work in China has also shown no difference in the prevalence of PPD in primiparous and secondiparous women [38].

## 4. Breastfeeding in the Prevention of PPD

More than ten years ago, Figueredo et al. [39], through a literature review, highlighted the presence of the first scientific evidence in favor of the potential protective role of breastfeeding against PPD. They illustrated the presence of specific hormonal and psychological mechanisms related to breastfeeding that are negatively associated with PPD [39]. Indeed, there is evidence not only of the possible dampening effect of neuro-endocrine responses to lactation-induced stress, especially by acting on basal diurnal cortisol levels [40,41,42,43,44,45], but also of the possible antidepressant action induced by lactogenic hormones such as oxytocin and prolactin [45,46]. Skin-to-skin contact prior to breastfeeding also contributes to this effect [44,45]. Indeed, recent scientific research has demonstrated that the maternal hypothalamic–pituitary–adrenal (HPA) axis undergoes adaptations during both pregnancy and lactation. These adaptations are likely to be beneficial in mitigating the potential negative effects of stress on the mother and, consequently, on her offspring.

A significant body of research has indicated that, during lactation, suckling the infant creates a neural stimulus that alters the circadian rhythm of the HPA axis, reducing stress responses. A reduction in the activity of corticotropin-releasing factor neurons in the paraventricular parvocellular nucleus has been observed, which is responsible for a reduction in noradrenergic input. This, in conjunction with the action of prolactin, appears to be responsible for the low stressor responses [40]. A study on the acute effects of lactation on the HPA axis and sympathetic–surrenal–medullary system responses to mental stress in lactating women observed that lactation in women, in contrast to what has been observed in some mouse models, does not result in a general containment of the HPA axis response to a psychosocial stressor. Instead, it appears to result in a short-term suppression of the cortisol response to mental stress [41]. Other research has also observed that lower basal diurnal cortisol secretion induced by lactation is more clearly observed in multiparous mothers. This is likely due to the fact that multiparous mothers are probably exposed to greater domestic demands and responsibilities. However, there are other factors influencing this secretion, such as socioeconomic status [43]. Handlin et al. [44] conducted a comprehensive analysis of the mechanisms underlying adrenocorticotropic hormone (ACTH) and cortisol release by mothers during a breastfeeding session, relating them to both maternal oxytocin levels and the duration of suckling and skin-to-skin contact prior to breastfeeding. This research demonstrated that breastfeeding is associated with a reduction in ACTH and cortisol levels, an effect that is further supported by skin-to-skin contact. Furthermore, a negative correlation was found between both ACTH and suckling duration and between ACTH and median oxytocin levels. Conversely, it was observed that cortisol levels correlated inversely with the duration of skin-to-skin contact preceding suckling. This led the authors to conclude that there is likely to be a partial dissociation between the mechanisms regulating ACTH and cortisol release [44].

Other possible favorable consequences of breastfeeding, inversely correlated with the onset of PPD, have been highlighted, such as the regulation of sleeping and waking patterns for mother and child, the improvement of the mother’s self-efficacy and her emotional involvement with the child, including through the reduction in the temperamental difficulties of the infant [39,45]. In this regard, the relationship between breastfeeding and maternal sleep has been the subject of various studies, indicating several benefits for breastfeeding mothers compared to those who use formula feeding. In fact, research has shown that breastfeeding mothers often experience better sleep quality and longer sleep durations than formula-feeding mothers. One study found that mothers who breastfeed tend to have deeper and more restorative sleep, possibly due to the hormonal influences of breastfeeding that promote relaxation and sleepiness [47]. Moreover, a time-use study in Australia highlighted that breastfeeding mothers may spend less time awake at night compared to formula-feeding mothers. This might be due to the convenience of breastfeeding, which eliminates the need to prepare bottles and allows for a quicker return to sleep after feeding the infant [48]. Another longitudinal study indicated that breastfeeding mothers maintained better sleep patterns over a more extended period postpartum. This was attributed to the more synchronized sleep–wake cycles between the breastfeeding mother and her infant, reducing disturbances in sleep continuity [47]. These findings suggest that breastfeeding has positive effects on maternal sleep, not only improving sleep quality and duration, but also fostering a more synchronized sleep routine with the infant, which can further enhance overall sleep efficiency. Specifically, sufficient sleep, a crucial aspect of maintaining health, is a common challenge for new mothers, persisting not only in the initial days but extending into the first few weeks and months [49,50]. The impact of sleep deprivation on maternal well-being can be so profound that it can lead to symptoms similar or correlated with postpartum depression, profoundly affecting the mother’s emotional well-being [50,51]. 

With regard to self-efficacy, in the postpartum period, it is correlated with health-seeking behaviors and inversely associated with maternal stress and depression [52,53]. Self-efficacy plays a crucial role in a woman’s ability to perform infant care and other parenting tasks, as it relies on her confidence [1,54,55]. Direct experience with a task or similar tasks has a strong influence on self-efficacy [56]. This is evidenced by the fact that multiparas have higher levels of self-efficacy than primiparas, and maternal self-efficacy increases as the postpartum period progresses [54]. In this regard, breastfeeding has also been shown to increase maternal self-efficacy, with higher levels of breastfeeding self-efficacy correlating with lower levels of PPD symptoms [57,58,59]. 

Infant temperament is another important factor influencing maternal self-efficacy during the postpartum period [60]. Women with difficult-to-soothe infants lose confidence in their ability to meet their infants’ needs and are more likely to report fewer attempts to soothe them [61]. Breastfeeding has also proved beneficial in this context. In fact, a study of 78 mothers, 31 of whom were depressed, and their children showed that mothers with PPD who had a stable breastfeeding pattern were less likely to have children with a highly reactive temperament [62]. Furthermore, research has indicated that breastfeeding may have a positive effect on reducing irritability and colic symptoms, as well as promoting longer periods of sleep. These factors have been shown to positively influence a child’s behavior. It has been hypothesized that these benefits may be attributable to the melatonin present in breast milk during the evening hours [63]. It was also shown that stable breastfeeding, even in depressed mothers, was correlated with more positive dyadic interactions [62].

However, sometimes reviews of the literature on this subject have shown little robust and conflicting data, potentially also due to the methodological limitations of the research conducted so far, with differences in measurements between studies and a lack of simultaneous analysis of the interacting effects of multiple hormones and their potential explanatory value in the connection between breastfeeding and pre- and postpartum depression [39,45]. 

A systematic literature review conducted in 2015 aimed at analyzing the effects of breastfeeding and maternal health found that a short duration of breastfeeding was associated with a higher risk of PPD [64]. In the same year, another systematic literature review demonstrated an association between PPD and shorter breastfeeding duration [65]. This aspect ties in with the review by Figueredo et al. [39] on the correlation between the HPA axis, oxytocin and PPD. Indeed, maternal psychological distress may compromise lactation. This is attributable to the delicate role of cortisol which, although it is a necessary cofactor for milk production, with implications for mammary gland cell differentiation, is maintained at low levels by lactation itself, probably to attenuate the reactivity of the maternal HPA axis to stress. However, maternal psychosocial distress and difficulties with lactation are possible causes of HPA axis disruption, with impaired milk production and secretion, which may interfere with oxytocin release from the pituitary posterior gland, leading to reduced milk ejection, and being responsible for reduced insulin secretion and sensitivity, hindering milk synthesis in lactocytes [45].

To date, the most recent data in the scientific literature seem to have dispelled any doubts, demonstrating that breastfeeding is crucial in reducing the risk of PPD. In fact, a recent systematic review of the literature showed that women who did not exclusively breastfeed were 89% more likely to have PPD [14]. A meta-analysis conducted in the same year, examining eight studies with a total of 18,570 participants, also found that breastfeeding was associated with a 14% reduction in the risk of PPD, with variations according to duration and exclusivity of breastfeeding. In fact, in the case of exclusive breastfeeding for more than one month, the risk reduction was 37%, while, for shorter durations, it was only 6%. With regard to exclusivity, on the other hand, the reduction was 53% when compared with no breastfeeding, while it was reduced to 8% when compared with mixed breastfeeding [66]. 

Furthermore, a recent cross-sectional study [67] conducted in Brazil on a sample of 287 women of puerperal age selected from two maternity hospitals, one public and one private, showed an association between greater maternal satisfaction with breastfeeding and the absence of PPD symptoms, supporting the delicate role of breastfeeding in the onset of PPD. This is further supported by the results of a recent systematic literature review [68] which showed that, while breastfeeding is associated with improved maternal mental health, it may be responsible for negative consequences if there are difficulties or discrepancies between expectations and actual experience. This led the authors to conclude on the necessity and importance of specifically investigating the breastfeeding experiences of women with mental health problems in order to help clinicians better tailor breastfeeding [68].

## 5. Support in the Postpartum Period

The self-care needs of women in the early postpartum period extend beyond physical health, encompassing crucial elements of emotional well-being. Many women reported experiencing high levels of sacrifice, often neglecting their own self-care needs to attend to the demands of their newborns [49,50,51,69,70,71]. Evidence in the literature points to a tendency for mothers to neglect universal, health-related self-care needs (such as sleep, feeding and pain management) in the early postpartum period in order to prioritize newborn care [49,50,51,69,70,71]. In addition, professional support, particularly in breastfeeding and infant care, is perceived as essential needs to enhancing parental confidence [69,71]. Also, the physical problems and pain resulting from breastfeeding are health needs that women must manage with the support of health personnel [49,69,70,71].

In addition, it is necessary for mothers to acquire skills to reorganize their daily routines already in the first weeks after delivery [72]. The literature underscores a significant gap, with guidelines and protocols primarily focusing on emotional support framed in terms of infant health rather than maternal well-being [73]. 

The omission of adequate attention to mothers’ mental well-being by healthcare professionals is deemed unacceptable, given that many women express dissatisfaction with postpartum care due to insufficient emotional support [74,75]. In this regard, an Australian study conducted in 2015 clearly showed that mothers reported substantial inadequacies in breastfeeding support, in hospital and at home, regardless of the type of hospital (public or private). In addition, some women complained of too early of a discharge, particularly from public facilities, while women from private facilities expressed more concern about post-discharge care, mainly due to a lack of follow-up. This lack of support was thus responsible, according to some mothers, for a negative effect on their psychological and emotional well-being [76]. A very recent qualitative study conducted in a Swiss university hospital exploring the perceived support needs of first-time parents in the postpartum period also highlighted the centrality of breastfeeding support. In fact, mothers had many questions regarding breastfeeding positions and how to breastfeed. In fact, although it is linked to happiness, confidence and successful motherhood, breastfeeding is also associated with feelings of distrust, anxiety and insecurity [51,69,77]. Moreover, learning to breastfeed varies from woman to woman, with some taking longer than others [71,77,78]. Furthermore, the importance of breastfeeding support in the prevention of PPD is even more evident given the correlation that has emerged in numerous research studies between breastfeeding and improved maternal self-efficacy [57,58,59]. Indeed, the ability to complete a challenging task has been shown to increase self-efficacy and the likelihood of persevering, even in the face of difficulties [56], with favorable conditions in reducing the risk of PPD onset. This is further supported by the correlation between a lower risk of PPD and breastfeeding satisfaction [67,68].

Differences in parental needs also emerged, with mothers needing more emotional support than fathers [79]. Poor postnatal support is also complained of in the UK, as revealed in a longitudinal qualitative descriptive study by McLeish et al. [80]. They first highlighted the paucity of information regarding primipara expectations of postnatal care and how these expectations correlate with their experiences. It also emerged that mothers’ satisfaction with postnatal care was primarily influenced, not by the extent to which their expectations were met, but by how their individual postnatal needs were met, emphasizing the need for and the importance of a rapid and responsive assessment of mothers’ actual needs [80]. Of note is the finding of a recent review of qualitative studies on breastfeeding support in association with the role of emotions and appraisal related to interactions with healthcare professionals in the UK [16]. This research found that breastfeeding ‘support’ could present challenges to maternal identity and thus emotional well-being, as many women had to manage interactions in order to avoid or minimize embarrassing emotions. Furthermore, the delicate role played by those providing breastfeeding support emerged as potentially responsible for both positive effects on the maternal emotional state through validation of their motherhood and negative effects by contributing to feelings of embarrassment, guilt or humiliation. This is also achieved by supporting mothers in their lactation choices, whether breastfeeding or formula feeding. In fact, this is the only way to validate their being mothers in any case, minimizing any feelings of inadequacy, conditions that help maternal emotional well-being. Therefore, staff involved in breastfeeding support need good emotional ‘antennae’ to ensure support and transition to motherhood. This led the authors to conclude that staff involved in postpartum care need to have good antennae, not only to provide proper support in the transition to motherhood, but also to understand whether the breastfeeding request actually hides other needs [16]. However, an updated review including 116 studies with a total of more than 98,816 mother–infant pairs found that support reduced the number of women discontinuing breastfeeding and thus also contributed to maternal emotional well-being [81].

**Figure 1:**
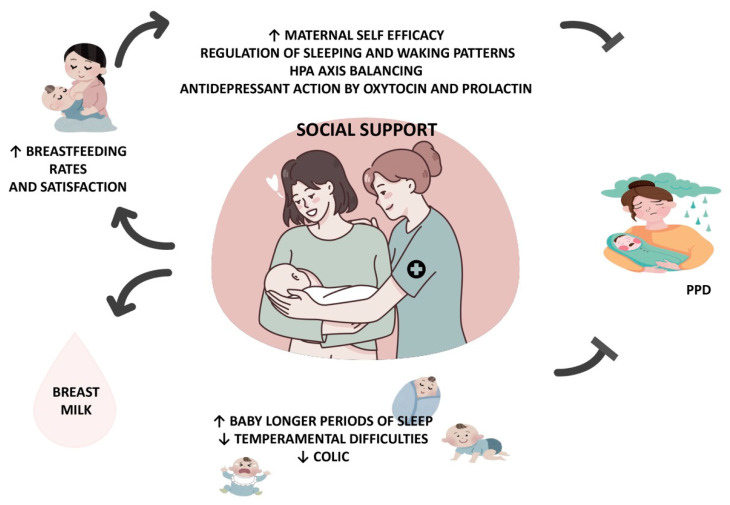
Relationship between healthcare and social support in the postpartum period, breastfeeding and the risk of PPD. Abbreviations: ↓ decrease, ↑ increase.

The relationship between healthcare and social support in the postpartum period, breastfeeding and the risk of PPD is illustrated in Figure 1.

Therefore, given the actual need for breastfeeding support by mothers, which emerges as one of the most important needs, often described as “the main thing” to learn in the early postpartum period [69,71,73,74,75,76,77,78,79,80,82], and recent data on the contribution of breastfeeding in reducing the risk of PPD [14,66,67,68], the importance of primary pediatric care is also outlined for implementing PPD screening [83,84]. This is supported by the difficulty, found in several studies, of connecting mothers with medical specialists for the treatment of PPD due to attitudinal, logistical and structural barriers [81]. Moreover, this is in agreement with the need and importance of pediatric primary care to directly address the growing pediatric mental health crisis [85,86]. It is indeed clear from the most recent data in the scientific literature that depression has become an increasingly important health and social problem. However, there is still an important need to improve mental health services and redefine existing measures. This can also be achieved by increasing the competence of doctors of other specialties in the diagnosis and treatment of this problem, whereas timely diagnosis and treatment are associated with faster remission and reduced relapse. Therefore, specific guidelines for primary care pediatric staff are useful in providing adequate support in the transition to motherhood.

**Figure 2:**
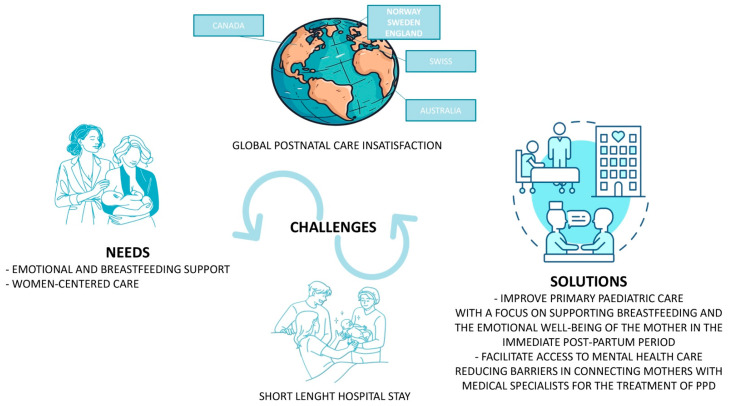
Post-birth needs, actual challenges and possible solutions.

Indeed, this literature review clearly shows, in high-income countries, low satisfaction in post-birth care support irrespective of the nation and its different health welfare mechanisms. This is combined with a widespread need for more emotional support and breastfeeding support, with particular emphasis on the demand for empathy and listening skills from health workers [16,69,73,74,75,76,77,78,79,80,82]. All high-income countries thus appear to share the same needs and issues, starting with studies conducted in Australia [74,76], from 2005, to the United Kingdom [16] and Switzerland [79], in 2022 and 2023, respectively. Northern Europe also experiences the same problems as reported by Swedish [75,77] and Norwegian [69] studies, and the same applies to North America [73]. The need for support after birth, the actual challenges and possible solutions to improve the current situation, which globally affect most high-income countries, is illustrated in Figure 2.

Neonatal nutrition can therefore play a dual role in this context. Indeed, primarily, it can regulate specific neurohormonal pathways that are protective of the mother’s emotional well-being and promote the psychological well-being of the dyad in the case of breastfeeding. However, adequate support for neonatal nutrition, regardless of the type of feeding, can also contribute to enhancing the uniqueness of each mother, in the absence of judgement, by counteracting any feelings of inadequacy, all of which are conditions inversely related to PPD [87,88].

## 6. Conclusions 

It is evident that health personnel must attend to the physical and emotional support needs of mothers during the postpartum period. The necessity for social support in the postpartum period, encompassing both the emotional dimension, including encouragement and support, and the instrumental dimension, which includes assistance in carrying out tasks, has been well documented. This fragment demonstrates that breastfeeding is one of the most crucial needs, often described as “the main task”, extending beyond the well-being of the infant to also affect maternal self-efficacy. Indeed, although women employ a variety of coping strategies to deal with the stressors of this period, self-efficacy, that is, the belief that one is able to complete tasks and achieve desired outcomes, has been shown to correlate positively with health-seeking behavior and is negatively associated with maternal stress and depression in the postpartum period. This has important positive repercussions in child development. Consequently, the promotion of robust social support and maternal self-efficacy in primary pediatric care is of paramount importance for a healthy postpartum period. It is similarly important to provide comprehensive support for women who have chosen to formula-feed.

In addition, it is crucial to recognize the inextricable link between the well-being of their mothers and the long-term health of infants and children. In light of these considerations, it is evident that health professionals share a responsibility to incorporate strategies that extend beyond conventional care practices. This involves actively and genuinely supporting women during the postpartum period, irrespective of the type of feeding undertaken, with the aim of fostering the development of individual capacities needed to successfully cope with the many challenges of postpartum care.

## Data Availability

Not applicable.
